# A rare but treatable cause of recurrent chest pain - Ictal chest pain

**DOI:** 10.1186/s12883-019-1575-0

**Published:** 2019-12-30

**Authors:** Ching Soong Khoo, Dongah Lee, Kang Min Park, Byung In Lee, Sung Eun Kim

**Affiliations:** 1 Department of Neurology, Haeundae Paik Hospital, Inje University College of Medicine, Haeundae-ro 875, Haeundae-gu, Busan, 612896 Republic of Korea; 2 Neurology Unit, Department of Medicine, Universiti Kebangsaan Malaysia Medical Centre, Kuala Lumpur, Malaysia

**Keywords:** Ictal chest pain, Chest pain, Epilepsy, Video-electroencephalogram

## Abstract

**Background:**

Chest pain as the primary manifestation of epilepsy is extremely rare and has only been reported once to date.

**Case presentation:**

We herein describe a 47-year-old woman with recurrent chest pain for 3 years. The cause of her chest pain remained elusive despite extensive investigations including comprehensive cardiac work-up. She was referred to the neurology clinic for one episode of confusion. Video-electroencephalographic monitoring detected unequivocal ictal changes during her habitual chest pain events. She has remained chest pain (seizure) free with a single antiseizure drug.

**Conclusions:**

This case underlines the importance of epilepsy as a rare yet treatable cause of recurrent chest pain. Further studies are required to determine the pathophysiology of ictal chest pain.

## Background

Chest pain is undoubtedly one of the most dreadful symptoms that anyone could endure. This is especially more distressing for patients when the underlying cause of chest pain is unclear. Ictal or epileptic chest pain is an extremely rare manifestation of epilepsy. We herein describe a case of ictal chest pain with favorable response to antiseizure therapy.

## Case presentation

A previously healthy 47-year-old woman presented with left-sided chest pain for almost 3 years. The chest pain was described as sharp and excruciating in nature with no radiation, which occurred initially once in every 3 months. It could happen any time of the day, mostly at night and each episode lasted about five to ten seconds. Emotional stress precipitated the pain. For the last 1 year, her symptom became more frequent. There were no other associated symptoms such as breathlessness, loss of consciousness or abnormal movements. Extensive investigations of her cardiovascular system including echocardiogram, coronary and chest computed tomograms were normal. She reported only one episode of abnormal behavior, which was why she was referred to our neurology clinic. It was described that she woke up in the bathroom in a confused state. The last thing she remembered was that she had been watching a television program in her bedroom.

**Fig. 1:**
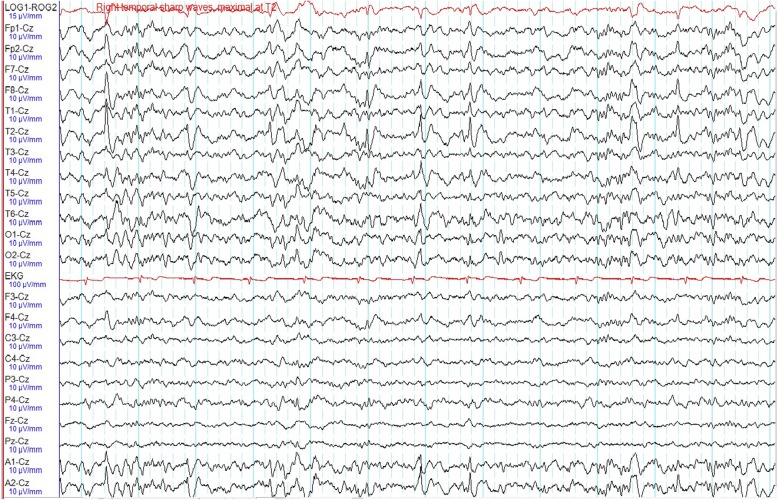
Interictal EEG shows sharp waves in the right temporal region, maximally at T2

**Fig. 2:**
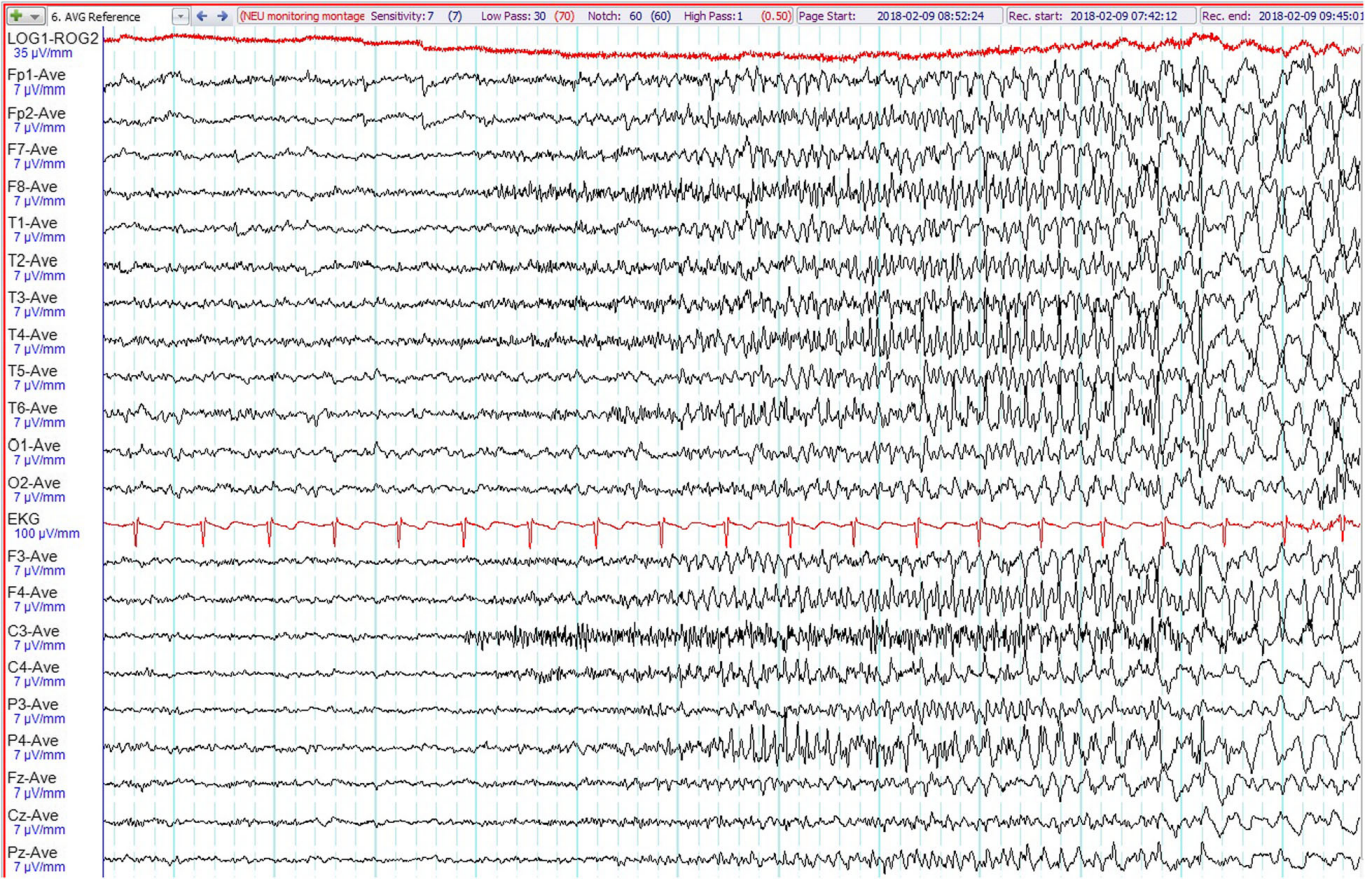
Ictal EEG reveals background attenuation, followed by fast rhythmic spiking from the right hemisphere

**Fig. 3:**
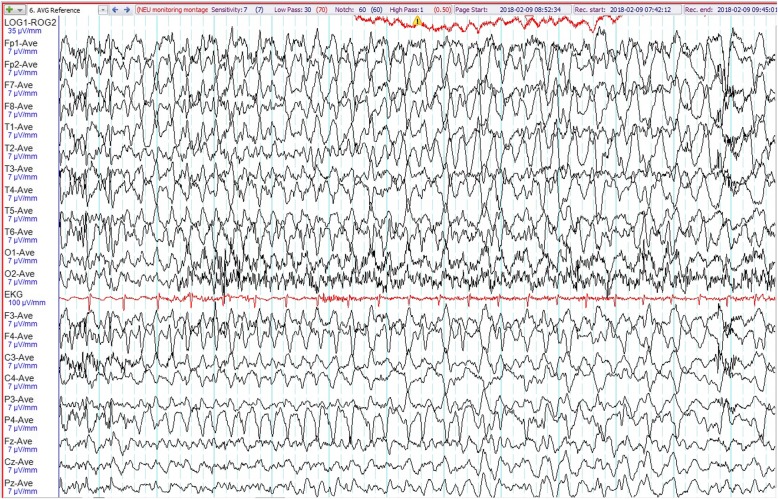
Continuation of ictal EEG shows evolving rhythmic 4 Hz delta activities

System review including her neurological examination was unremarkable. Her vital signs were as follows: blood pressure 100/60 mmHg, pulse rate 68 beats per minute and temperature 36.6 °C. Blood results revealed normal renal function with no electrolyte imbalances. Random blood glucose was 122 mg/dL. Video-electroencephalographic (EEG) recording captured three habitual chest pain episodes associated with definite ictal EEG changes. During all these events, she was seen suddenly waking up from the bed and touching her chest wall moaning in pain. Prior to this, she had discomfort in her chest and felt nauseous. In one of these attacks, she had a blank stare for about 3 seconds before the chest pain. This habitual event lasted about 10 seconds. We recorded no stiffening, convulsions or any other abnormal movements. Electrocardiogram revealed sinus rhythm with no ischemic changes or sinister arrhythmias during these events. During all these attacks, there were no significant changes in blood pressure, pulse rate, electrolyte and glucose levels. Cardiac enzymes were repeatedly within normal limits. Intermittent right temporal sharp waves (maximally at T2) were seen in the interictal EEG tracing (Fig. 1). During the attack, background attenuation was seen, which was followed by fast rhythmic spikes from the right hemisphere (Fig. 2). They gradually evolved into 4 hertz (Hz) delta activities that increased in amplitude (Fig. 3). Video EEG recording of her attack is in Additional file 1. A 3-T brain magnetic resonance imaging (MRI) revealed no appreciable lesions. A diagnosis of epilepsy was established and she has been chest pain (seizure) free with a daily dose of lamotrigine at 200 mg for the last 1 year. Follow-up EEG was normal.

## Discussion and conclusions

Ictal or epileptic pain as a subjective symptom or aura has not received much attention. Despite its reported frequency varying from 0.2 to 23.6% [1–3], the exact pathophysiology of pain is yet to be elucidated. This highly variable frequency could possibly be explained by different perceptions about pain due to cultural and emotional differences [4]. Moreover, ictal pain rarely occurs in isolation, and thus it might be under-represented or under-reported.

Epileptic pain is commonly reported to occur in one side (or a part) of the body, head (cephalic) or abdomen. Unilateral painful seizures are of lateralizing value, with the seizure origin arising from the contralateral hemisphere to the pain [5]. Frontal [5] and parietal lobes [4, 5] are frequently involved. Asadi-Pooya et al. were however unable to demonstrate localizing or lateralizing value of ictal pain in his 12-year retrospective study on 5133 subjects. Ictal headache is frequently associated with focal epilepsy [6, 7]. In one study, epileptic headache is reported to be more likely ipsilateral to the seizure onset in temporal lobe epilepsy than extratemporal lobe epilepsy [8]. Epileptic abdominal pain is usually confined to the periumbilical region or upper abdomen and it rarely spreads [9, 10]. It commonly happens in children or young adolescents and responds well to antiseizure drugs. In adult patients, temporal lobe is thought to be involved in abdominal pain [5].

The role of cortical sensation in pain perception has long been investigated [5, 11]. The major areas involved are the primary sensory area in the postcentral gyrus, secondary sensor area in the upper part of the sylvian fissure and supplementary sensory area in the mesial frontal region [12]. Ictal intracranial tracings recorded ictal onsets with pain from the parietal operculum, medial parietal lobe and inferior parietal lobule in one study [4]. The thalamus is postulated to be another crucial structure in the interplay between pain and seizures. It has extensive networks connecting many cortices, which include the insular, parietal and temporal cortices via the thalamocortical circuit [13]. It also plays a critical role in pain control through the spinothalamic tract. Epileptic pain due to thalamic syndrome has been suggested as a result of interruption of the thalamocortical interplay [14]. The involvement of a multitude of brain structures and areas in various studies suggests that pain perception has a wide and complex network.

Ictal chest pain has not been documented in many large scale studies, except in one, in which chest pain was encountered in three patients with psychogenic non-epileptic seizures (PNES). In this study, PNES was the most common cause of ictal pain [2]. Pure epileptic angina as a presenting complaint was only reported by Sureshbabu et al. in a 14-year-old boy [15]. Left centroparietal ictal rhythm was identified during the chest pain and the patient responded well to carbamazepine. It was posited by the authors that the mesial parietal lobe and cingulate cortex were responsible for ictal chest pain, given the paracentral distribution. Involvement in these two areas have been reported to cause sensory manifestations [4, 16]. We hypothesized that the likely involved areas responsible for her seizures were the right parietal operculum and insula. Viscerosensory auras such as chest discomfort, nausea, epigastric sensation have been reported in insular seizures [17]. Such symptoms might be difficult to be differentiated from those of temporal lobe seizures on clinical grounds as they both have bidirectional interconnections. The scalp EEG of our patient revealed sharp waves in the anterior temporal region, which have been described in insular epilepsy too. The insula is an important area responsible for autonomic control, with the right insula being more dominant in the sympathetic nervous system regulation [17]. This could further support right insula as the likely epileptogenic focus in our case. Both our cases had no lesions detected on the MRI and had a favourable outcome with antiseizure medications. Table 1 illustrated the differences in both our patient and the previously described case.

**Table 1:** Differences in our case and the previously reported case

	Sureshbabu et al.	Khoo et al.
Age of onset (years)	14	44
Gender	Male	Female
Chest pain location	Left-sided	Left-sided
Duration	A few seconds to half a minute	Five to ten seconds
Associated symptoms	No	One episode of confusion
Interictal EEG	Spike-and-wave discharges in the left frontocentral region	Sharp waves in the right temporal region
Ictal EEG (onset)	Left parasagittal region	Right hemisphere
Intracranial recording MRI	Not performed Normal (1.5-T)	Not performed Normal (3-T)
Antiseizure	Carbamazepine 400 mg (daily)	Lamotrigine 200 mg (daily)
Outcome	Seizure free	Seizure free

Abbreviations: *EEG* Electroencephalogram, *MRI* Magnetic resonance imaging

We were unable to exactly localize the epileptogenic zones in both our cases. However, unequivocal ictal EEG changes during the habitual events and remarkable response to antiseizure drugs firmly established our diagnosis. Further prospective studies are encouraged to yield more robust conclusions on the mechanism of ictal chest pain.

## Conclusions

Ictal chest pain is extremely rare as an isolated epileptic manifestation. For individuals with unexplained recurrent chest pain, detailed investigation such as video-telemetry might be warranted to avoid treatment delay in potentially treatable conditions, such as epilepsy.

## Supplementary information

**Additional file 1.** The patient suddenly felt uncomfortable and called for help. She sat up quickly, put her palms over her chest and moaned in pain. During this episode, fast rhythmic spikes from the right hemisphere were seen. They gradually evolved into 3-4 hertz (Hz) delta activities that increased in amplitude.

## Data Availability

Available upon request to the corresponding author.

## Abbreviations

EEG: Electroencephalogram
Hz: Hertz
MRI: Magnetic resonance imaging
PNES: Psychogenic non-epileptic seizures
